# Misoprostol Abortion: Ultrasonography versus Beta-hCG Testing for Verification of Effectiveness

**DOI:** 10.12669/pjms.296.3361

**Published:** 2013

**Authors:** Fariba Behnamfar, Mehrdad Mahdian, Fereshteh Rahimi, Mansoureh Samimi

**Affiliations:** 1 Fariba Behnamfar, Associate Professor, Department of Obstetrics and Gynecology Isfahan University of Medical Sciences, Isfahan, Iran.; 2 Mehrdad Mahdian, Trauma Research Center, Kashan University of Medical Sciences, Kashan, Iran.; 3 Fereshteh Rahimi, Department of Obstetrics and Gynecology, Kashan University of Medical Sciences, Kashan, Iran.; 4 Mansoureh Samimi, Assistant Professor, Department of Obstetrics and Gynecology, Kashan University of Medical Sciences, Kashan, Iran.

**Keywords:** Medical abortion, Misoprostol, hCG, Ultrasound, Verification of expulsion

## Abstract

***Background and Objective:*** Miscarriage is a common complication of early pregnancy with medical and psychological consequences. Dilation and Curettage are considered as two standard caring ways for early pregnancy failure. Alternatively misoprostol has been used as a single agent for termination of early pregnancy. Aim of the present study was to compare the usefulness of serum β-hCG measurement and ultrasound examination to predict complete abortion after medical induction.

***Methods:*** There were one hundred and thirty three patients experiencing missed abortion or blighted ovum. Ultrasound examination and serum β-hCG test were performed before treatment and during follow-up in all these patients.

***Results:*** Treatment was successful without any need for surgical intervention in 92.4% of the cases. Both methods could verify the complete abortion among all the patients at the end of the study (4^th^ week). Kappa agreement coefficient for the two methods of diagnosis was 0.327 (P < 0.5).

***Conclusion:*** Based on our results, β- hCG is as effective as ultrasound in confirming a successful medically induced abortion in early pregnancy, but it should be used as supplements to clinical assessments.

## INTRODUCTION

Early pregnancy failure includes 15–20% of all pregnancies.^1^ Of the all recognized pregnancies, 15–20% end spontaneously in the first and second trimester.^2^ Miscarriage is a common complication of early pregnancy that can have both medical and psychological consequences like depression and anxiety.^3^ Incomplete abortions can be managed expectantly, surgically and medically.^4^ Dilation and curettage have been used for managing early pregnancy failure for more than half a century for the sake of high-quality medical care. Expectant management also works well; however, it is sometimes less desirable to women (and some providers) who may prefer immediate treatment.^4^ Medical abortion is an alternative treatment being used widely for 30 years. It may be less costly than surgical treatment and consequently due to its lower complications most patients prefer it.^1^

Misoprostol is a stable synthetic prostaglandin E1 analogue that has been used for the prevention of gastric ulcers induced by non-steroidal anti-inflammatory drugs. Since like other prostaglandins, this agent increases uterine tone^5^, it has been used off-label for cervical priming or ripening in medical abortion, induction of labor, and prevention of postpartum hemorrhage. Researchers have reported the high efficacy and safety of misoprostol used as a single agent for the termination of early pregnancy failure. 200–800 μg of vaginal misoprostol is successful for the treatment of early pregnancy failure up to 60–80%.^6^^-^^10^

Confirming a complete medical abortion at the follow-up visit is an important component of completing a medical abortion. Three methods can be employed to confirm a complete abortion: (1) sonogram examination documenting an empty uterus, (2) finding a 50% decrease per day in serial serum hCG levels, and (3) observing the products of conception during the in-office misoprostol visit.^11^ The use of ultrasound to determine the outcome of medical abortion and possible need for surgical intervention clearly requires knowledge of the ultrasound finding following medical abortion. In addition, ultrasound at follow-up is sometimes difficult to interpret.^12^

Monitoring serial hCGs is a very reliable and safe alternative to sonogram. Measuring hCG is also reliable in detecting a persistent pregnancy or incomplete expulsion even in the case of extra uterine. Successful expulsions were consistent with a marked decline in hCG value at follow-up. Measuring hCG can easily be done in most places and might be more convenient for women as compared to an ultrasound examination.

Furthermore there is no need to have it performed in the institution that performed the medical abortion.^2^ Series of studies have compared reliability and safety of ultrasound and measurement of hCG to confirm complete abortion. Some studies have concluded that measurement of serial hCG is preferred than ultrasound.^12^ Some studies showed similar efficacy^13^ and some emphasized on supplementation of these measures with clinical assessment.^14^^,^^15^

The aim of the present study was to compare the usefulness of serum β-hCG measurement and ultrasound examination to predict completeness of abortion after medical induction.

## METHODS

One hundred and forty four women with diagnosis of missed abortion or blighted ovum [confirmed by vaginal ultrasound (Medison 8000, vaginal probe 7.5 MHz)] with a gestational age less than 12 weeks and a crown-rump length (CRL) of 10 mm and a positive β-hCG test who were candidate of medical abortion after obtaining approval from our institutional review board (IRB) and written consent, were enrolled the study during 2010. Levels of β-hCG were measured by ELISA test using a commercial kit (Immunotech, Beckman Counter Co, Czech Republic). Exclusion criteria were fever (T>38˚C), leukocytosis, thrombocytopenia, sever uncontrollable bleeding, anemia, mole hydatiform and septic abortion. The patients received 800 mg of misoprostol vaginally at the hospital. After 24 hours, if bleeding had not started at that time or it was lower on the first day of menstruation, a second dose of 800 μg misoprostol was orally given.

In cases with expulsion of pregnancy products, placenta was checked exactly by the physician. Women who remained undelivered by 24 hours after the last dose (receiving totally 1600µg), underwent surgical intervention and were excluded from the study. All the women with confirmed expulsion of pregnancy products in hospital, and regardless of presence or absence of remained products, combined contraceptive (LD) were immediately administered orally per day up to 21 days. The first follow- up to assess the treatment outcome was performed on the day 15 after expulsion. The ultrasound examination and β-hCG testing were repeated at this time. In cases with negative or declined β-hCG more than 80% and empty uterine in ultrasound (no remained material or endometrial thickness<15 mm^14^^,^^16^^,^^17^) follow-up was terminated. If not, the women were advised to continue using contraceptive until the 4^th^ week and then β-hCG test and ultrasound were repeated.

Finally, in cases with negative β-hCG and without remained products in uterine or endometrial thickness less than 15 mm in ultrasound, the treatment was regarded as successful and the follow-up was completed. Otherwise, the patient was referred for surgical treatment and excluded from the study. The β-hCG levels at follow-up are given as the percentage of the value before the treatment and are presented as mean and (S.D.). Measurements of endometrial thickness are given in mm and presented as mean and (S.D). The Sample size was calculated (minimum 60 cases in each group) using the method of non-inferiority calculation and based on a similar study^15^ in which ultrasonography had a verification of 89.7% successful medically induced abortion in early pregnancy, considering confidence interval of 95%, power of 90% and maximum acceptable difference of 0.2.

The data were analyzed using the Levene test and *t*-test. Agreement regarding the two variables (β-hCG and ultrasound) was performed using the kappa coefficient. The level of statistical significance was set at *P* < 0.05.

## RESULTS

A total of 144 women were initially enrolled in the trial (age range=15-45 years), but 11 cases withdrew from the study due to surgical intervention. Rest of the patients (133 cases) completed the treatment and returned for follow-up. Women’s characteristics such as maternal age, gravidity, parity, pregnancy duration prior to abortion and body mass index were similar in the two groups. Of the 133 remained women, the most ultrasound finding before treatment was gestational sac (41.7%) and gestational sac with yolk sac (28.5%). There was a significantly higher rate of complete abortion (56.3%) among the women who received the second dose of misoprostol (1600 mcg) comparing with those receiving 800 mcg of the drug (43.7%) (P < 0.05).

**Table-I:** Comparing ultrasound and β-hCG measurements to verify the effectiveness of the treatment

*Diagnosis Method of Complete abortion*		*Ultrasound*		*Sum*	*Kappa Coefficient*
		*After 2 weeks (%)*	*After 4 weeks (%)*		
β-hCG	After 2 weeks (%)	79(98.75)	37(69.8)	116 (87.2)	0.327
	After 4 weeks (%)	1 (1.25)	16 (30.2)	17 (12.8)	
Sum		80 (100)	53 (100)	133 (100)	

**Table-II:** Comparing post-induction ultrasound and β-hCG measurements to verify the effectiveness of the treatment based on history of pain.

*Variables*	*With Pain*		*Without Pain*	
	*β-hCG* * (%)*	*Ultrasound*	*β-hCG* * (%)*	*Ultrasound*
Diagnosis at 2^nd^ week	24 (96)	21 (84)	92 (85.2)	59 (54.6)
Diagnosis at 4^th^ week	1(4)	4 (16)	16 (14.8)	49 (54.4)
Sum	25 (100)	25 (100)	108(100)	108 (100)

The treatment was successful without any need for surgical intervention in 92.4% of the cases. Among the 133 cases, 116 cases (87.2%) based on β-hCG and 80 cases (60.15%) based on ultrasound were diagnosed with complete abortion in the second week of treatment. In addition, of the 80 cases, 79 (98.75%) were also diagnosed by β-hCG, while of the 116 cases, 79 (68.1%) were diagnosed by ultrasound as well. Kappa agreement coefficient for the two methods of diagnoses was 0.327 (P < 0.5). Both methods could verify complete abortion in all the patients at the end of the study (4^th^ week) (Table-I). β-hCG was more successful than ultrasound for the diagnosis of complete abortion at the second week in cases that have had pain as a symptom (Table-II). Of the 11 cases that were excluded from the study, two patients were hospitalized due to heavy bleeding. Both of them required surgical intervention after uncontrolled bleeding and the 8 remained cases did not expel products of conception after 24 hours of second dose of misoprostol and underwent surgical evacuation.

## DISCUSSION

The results of this study showed that administrating misoprostol at a dose of 800 to 1600 mcg vaginally is an effective non-surgical method of treatment for evacuation of the uterus after abortion and measuring serum β-hCG is as effective as ultrasound to confirm a successful medically induced abortion in early pregnancy.

In the present study, the success rate after using vaginal misoprostol was 92.4%. Ngoc et al. (2004) found 92.9% success rate after administration of 800 mcg misoprostol.^2^ Fiala et al. (2003) reported a 98.2% success rate in their study using mifeprostone 600 mg and misoprostol 400 mcg orally followed 3 hours later by a second dose of 400 mcg misoprostol if the woman had not started to bleed.^12^ Behrashi and Mahdian (2008) have shown the 86.7% success rate for vaginal misoprostol to second trimester pregnancy termination using it up to 1200 mcg.^18^ Our finding is in agreement with Ngoc's findings.

Slightly higher effectiveness in Fiala's study may be due to difference in the used drugs and/or different methods of administration. Synergistic effect of mifeprostone with misoprostol may be another reason. Using different dosages in the present study compared to Behrashi's report (1600 vs 1200mcg) may be the reason of higher success rate in our study. In the present study, β-hCG measurement could verify complete abortion during the second week in 87.2% of the cases, however, ultrasound could confirm it in 60.5% of the women. In Fiala's study these values at the second week for β-hCG and ultrasound were 98.5% and 89.8%, respectively.^12^ There is a similarity between our findings and Fiala's reports regarding β-hCG measurements but, differences in the results of ultrasound examination may be due to ultrasound devices dissimilarity (may be older devices with less sensitivity in our center) and sonographer skills leading to wrong diagnoses.

Clark et al. conducted a study to determine the safety and efficacy of providing medication abortion in a primary care site without routine use of pre- and post-procedure transvaginal sonography. In their study all the patients were intended to be followed up with serum hCG testing pre- and post-treatment. They used sonography only as needed for specific indications such as no successful medical abortion, no hCG decline by at least 80% or in cases with uncertain history. They concluded using clinical protocol based on obtaining pre- and post-treatment serum hCG measurement with sonograms only when indicated had similar outcome to a protocol that used mandatory pre- and post-sonograms.^13^

El-baradie et al. (2008) showed that measurement of serum β- hCG and ultrasound are clinically useful measures for predicting late failure of medical abortion, but should be used as supplements to clinical measurements.^14^ Fielding et al. (2002) based on their study concluded that if clinicians monitor hCG levels to identify any ectopic or continuing pregnancies, medical abortion can be safely performed without sonography.^11^ Although it is expected that bleeding may continue for too long with medical abortion^17^, the duration of bleeding in the present study was 2.6 days (SD=2.1) and mostly with spotting. Short bleeding may be due to use of OCP by the patients in our study.

In conclusion, based on our results, measuring β- hCG is as effective as ultrasound to confirm a successful medically induced abortion in early pregnancy, but should be used as supplements to clinical assessments.
